# Considering Animals: Contemporary Studies in Human-Animal Relations

**DOI:** 10.3390/ani2030377

**Published:** 2012-08-22

**Authors:** Siobhan O’Sullivan

**Affiliations:** School of Social and Political Sciences, University of Melbourne, Parkville, VIC 3010, Australia; E-Mail: siobhano@unimelb.edu.au

In 2005 a small group of academics gathered at the University of Western Australia for a modest yet highly significant interdisciplinary conference focused on scholarship in the emerging field of human-animal studies. A critical mass of academics from the University of Tasmania attended that first conference and pledged to host a second human-animal studies conference two years later. True to their word a second human-animal studies conference was held in Hobart, Australia, in 2007. The organisers called the second conference “Considering Animals” and the book under review here is a compilation of papers presented at that conference.

**Figure 1:**
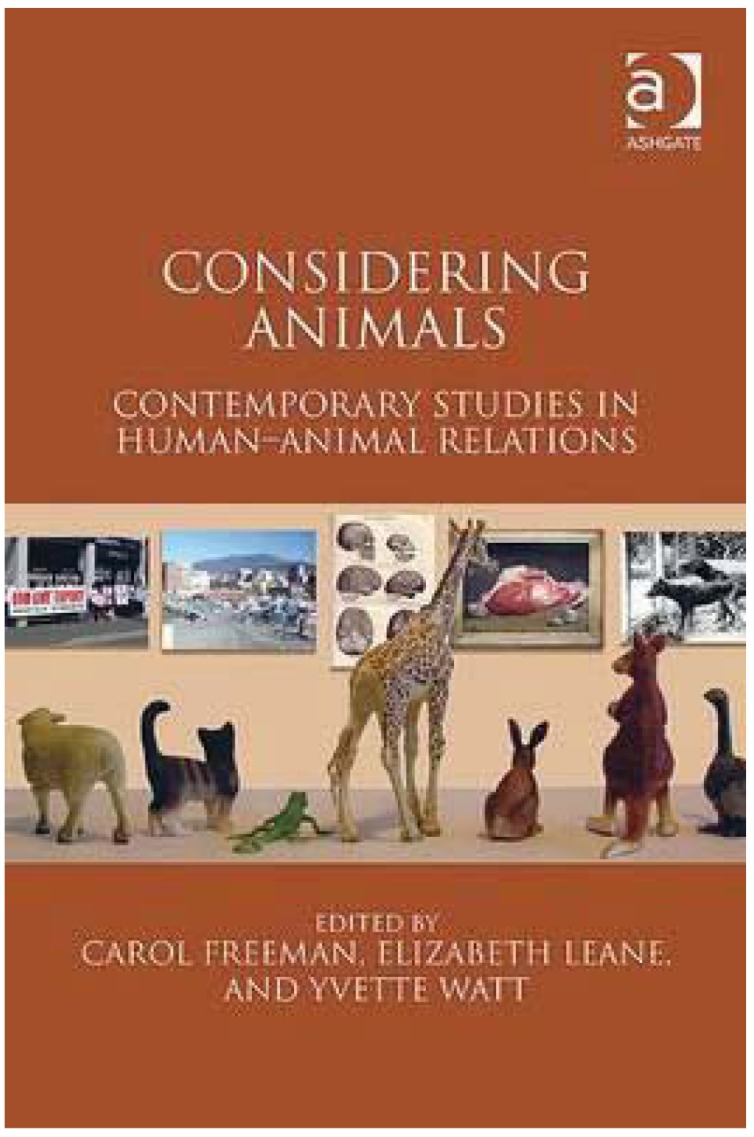
Dust jacket of Considering Animals.

The first striking feature of the book *Considering Animals* (hardback version), is the artwork on the dust jacket (Figure 1). While some may not pay a book’s dust jacket much heed, I view *Considering Animals* stunning cover-art as quite a coup. In an age of publishing rationalisation and belt-tightening, I imagine that the editors must have fought hard for permission to display a colour image on the book’s cover; and for the inclusion of such a large number of pictures throughout the book. If this is the case, then their persistence paid off. Not only is Yvette Watt’s cover-art beautiful and thought provoking in and of itself, it also serves to remind readers that this book is dealing with a highly interdisciplinary field of academic inquiry. Human-animal studies is not only about words. It is about images, representation, art and interpretation. One of the most noteworthy features of the bi-annual Australian Animal Studies Group, and the Minding Animals, conferences is the extent to which visual and other creative artists contribute to the field. With the use of such powerful cover-art the editors give effect to the contribution made by creative arts to the emerging discipline of human-animal studies.

The book opens with a forward by well-known ecologist Marc Becoff and an introduction by two of the book’s editors: Carol Freeman and Elizabeth Leane. The remainder of the book consists of 14 papers by (often prominent) academics, all of who presented at the 2007 University of Tasmania “Considering Animals” conference. 

Compilations of this nature have largely fallen out of favour in the competitive world of academic book publishing. *Considering Animals* demonstrates why collected works originating at conference are so difficult to edit. Bringing a coherent structure to a book that consists of papers by disparate scholars, each with their own assumptions concerning style, focus and emphasis is always a big challenge. In the case of a book concerned with human-animal studies the challenge is ten-fold greater because the field truly is profoundly interdisciplinary with the only unifying feature being an interest in the nonhuman animal. The editors therefore made a bold effort at bringing structure to the book by organizing it into three parts titled: Image; Ethics; and Agency. However, the groupings appear to me to be rather arbitrary. For example, I don’t know that any of the authors included in the Ethics section are actually ethicists. I am also not sure that what they write about is any more or less ethics centred than any other paper in the collection. The book’s organisation left me wondering whether the editors might have been just as well to divide the book into Part I, Part II and so on. 

Given the highly diverse nature of *Considering Animals* the reader is left to consider each contribution as a stand-alone paper based on its own merit and importance. As a political scientist, when faced with a collection of human-animal studies papers I tend to head straight for anything authored by a fellow political scientist, or legal studies scholar. There is a deficit of such papers in this book. That is not the fault of the editors, but rather a reflection of the small contribution political scientists are making to the field generally. Yet while there is nothing in the book that speaks to me directly in a disciplinary sense, I did find some fascinating papers that have informed my thinking and which I will use in my own scholarship. Kay Milton’s paper entitled ‘Possum Magic, Possum Menace’ was particularly engaging. Milton is interested in the mechanisms by which certain animals might be considered adorable in one context and loathsome in another. She writes:

*This essay compares the representation of possums in Australia and New Zealand in order to examine how New Zealand culture demonises possums, thereby overriding or ignoring any appeal their cuteness might have (p. 67). *

Milton looks to cultural norms and the socialization process to understand the phenomenon and concludes that:

*Cultural priorities determine whether animals are in the wrong place (possums in New Zealand, possums in Australian roof space) or the right place (possum in the Australian bush) and shape their treatment by human society (p. 77). *

While Milton’s essay is an example of an unexpected treasure that happens to speak to a recent research interest of mine, I nonetheless found many of the other essays engaging, instructive and thought provoking. For example, Jonathon Balcombe’s paper challenges the academic emphasis placed on the study of pain, at the expense of a focus on the enjoyment of pleasure; while Yvette Watt offers a very personal insight into the relationship between art, artists, and animal activism. 

Perhaps fittingly, the book’s final word goes to Tim Low who was the 2007 “Considering Animals” keynote. Low’s essay is titled ‘When is Nature not?’ and I must admit that it was my favorite essay of the book. In a discussion about the complexities of climate change and the role many different types of life play in our evolving environment, I found observations such as:

*“If killer ants had risen to world domination they would not have set aside safe areas for other species, nor devised laws to protect them” (p. 203).*

and:

*“We should be able to talk about seabirds “polluting” island soil, but the word “pollution” is almost never applied to nonhuman waste, and “erosion” is seldom applied to animal impacts” (pp. 203–204).*

highly thought provoking. In additional to having an interesting perspective on the world around us, Low is a highly skilled author and his craftsmanship as a writer shines thought the essay.

Because *Considering Animals* is so diverse in scope it is unlikely that every essay will speak to every reader. However, all human-animal studies scholars will find something of interest for them and I expect the book will find a home on the bookcases of many of my human-animal studies scholar colleagues in Australia and around the world. 

